# Chemical-Induced Read-Through at Premature Termination Codons Determined by a Rapid Dual-Fluorescence System Based on *S*. *cerevisiae*

**DOI:** 10.1371/journal.pone.0154260

**Published:** 2016-04-27

**Authors:** Emiliano Altamura, Monica Borgatti, Alessia Finotti, Jessica Gasparello, Roberto Gambari, Mariangela Spinelli, Rosa Castaldo, Nicola Altamura

**Affiliations:** 1 Chemistry Department, University of Bari, Bari, Italy; 2 Department of Life Sciences and Biotechnology, Biochemistry and Molecular Biology Section, University of Ferrara, Ferrara, Italy; 3 Institute of Biomembranes and Bioenergetics, National Researches Council, Bari, Italy; German Cancer Research Center, GERMANY

## Abstract

Nonsense mutations generate in-frame stop codons in mRNA leading to a premature arrest of translation. Functional consequences of premature termination codons (PTCs) include the synthesis of truncated proteins with loss of protein function causing severe inherited or acquired diseases. A therapeutic approach has been recently developed that is based on the use of chemical agents with the ability to suppress PTCs (read-through) restoring the synthesis of a functional full-length protein. Research interest for compounds able to induce read-through requires an efficient high throughput large scale screening system. We present a rapid, sensitive and quantitative method based on a dual-fluorescence reporter expressed in the yeast *Saccharomyces cerevisiae* to monitor and quantitate read-through at PTCs. We have shown that our novel system works equally well in detecting read-through at all three PTCs UGA, UAG and UAA.

## Introduction

It is estimated that at least 11% of all known inherited genetic diseases are associated with nonsense mutations that generate premature termination codons (PTCs). In-frame UGA, UAG and UAA in mRNA promote a premature arrest of translation leading to the synthesis of truncated, often un-functional or aberrant, proteins that result in pathological phenotypes. The presence of PTCs has been found in many diseases, including β-thalassemia, cystic fibrosis (CF), Duchenne muscular dystrophy (DMD), ataxia telangiectasia, Usher syndrome, Hurler syndrome and several types of cancer [1, 2]. To date there is no genetic therapy available for these disorders. Nonsense mutations activate nonsense-mediated mRNA decay (NMD) a process that specifically recognizes and degrades PTCs-containing mRNA and that could add difficulties in the efforts to alleviate the disease phenotype [3, 4].

Suppression therapy, based on chemical-induction of suppression at PTCs (read-through) but not at the natural stop codon has recently been developed [5]. Suppression of PTCs mediated by drugs restores translation elongation and stabilizes PTC-mRNA whose availability could be enhanced by attenuation of NMD [6, 7]. Two classes of compounds have been so far described as read-through inducers. The first class is composed by aminoglycoside antibiotics such as geneticin (G418) and gentamicin. These compounds have been demonstrated to be efficient in mediating read-through leading to the re-synthesis of a functional full-length product in *in vitro* studies as well as *in vivo* mouse models, in Duchenne muscular dystrophy (DMD), cystic fibrosis (CF), hemophilia, β-thalassemia and other non hereditary diseases, such as colon cancer [1, 2, 8, 9]. Clinical trials imply only a small number of patients with nonsense mutations are expected to benefit from gentamicin treatment whilst long-term treatment with aminoglycosides has been shown to produce severe nephrotoxic or ototoxic effects [10, 11]. A rational design strategy to provide synthetic aminoglycoside derivatives with increased read-through efficiency and decreased ototoxicity is required [12–14]. NB84 was recently demonstrated to be effective in moderating disease progression in a long-term suppression therapy [15].

The second class of read-through molecules is represented by nonaminoglycoside compounds, such as PTC124 (Ataluren), identified in a high-throughput screening (HTS) based on a luciferase reporter assay. PTC124 is an oxadiazole compound that has no antibiotic properties and was shown to be safe in pre-clinical trials. However its efficiency in mediating read-through has been questioned because (a) it was found to interact directly with firefly luciferase [16, 17] and (b) displayed with no read-through efficacy in a comparative assay with G418 that instead resulted active across multiple *in vitro* reporter assays [18]. PTC124 was demonstrated to be effective in mediating read-through in various studies [1] although it displays a selective activity for the UGA codon [19]. An alternate luciferase-independent HTS assay (PTT-ELISA) was developed that is based on an *in vitro* transcription and translation system driven by a plasmid containing a portion of the sequence of the ATM gene with a TGA C mutation [20]. By using this screening system a new series of small compounds were identified that induced read-through at all three types of nonsense mutation. Among these, RTC13 and RTC14 induced read-through at nonsense mutations in both the ATM and dystrophin genes [20]. In a second series of studies two other nonaminoglycosides compounds, GJ071 and GJ072, were identified and confirmed to be effective in mediating read-through in cells derived from ataxia telangiectasia (A-T) patients with three different types of nonsense mutation in the ATM gene [21]. Another nonaminoglycoside compound, Amlexanox, was recently identified as an inhibitor of NMD in a dedicated screening of a library of 1200 marketed drugs and found to be also able to induce read-through [22]. Most recently a novel class of natural compounds, analogues of (+)-negamycin, were discovered possessing a selective eukaryotic read-through ability that do not display antimicrobial activity [23]. Despite the fact some potential therapeutic molecules are already available, so far clinical data have been below expectations. Novel safe and efficient read-through molecules and repurposed drugs for therapeutic approaches to PTC-associated diseases are required.

Several reporter systems have been so far developed to facilitate the detection of read-through activity based on high throughput screening (HTS) of small compounds. These include dual enzyme reporters such as β-galactosidase-luciferase [23, 24] and dual luciferase [25] or the protein transcription-translation (PTT)–enzyme-linked immunosorbent assay (ELISA) [20, 21]. Enzymatic read-through assay systems have also been developed in *Saccharomyces cerevisiae* and the dual luciferase reporter system has been adapted for the expression in yeast [26–28]. Although powerful, these reporter systems are expensive and require cell lysis, reagents and time-consuming manipulations.

Here we have described a rapid, sensitive and inexpensive method based on dual-fluorescence reporters expressed in the yeast *S*. *cerevisiae* to monitor and quantitate read-through at PTCs. This novel system works equally well in detecting read through at all three PTCs UGA, UAG and UAA.

## Results

### Construction of a dual fluorescence reporter system for monitoring read-through in yeast

**Fig 1:**
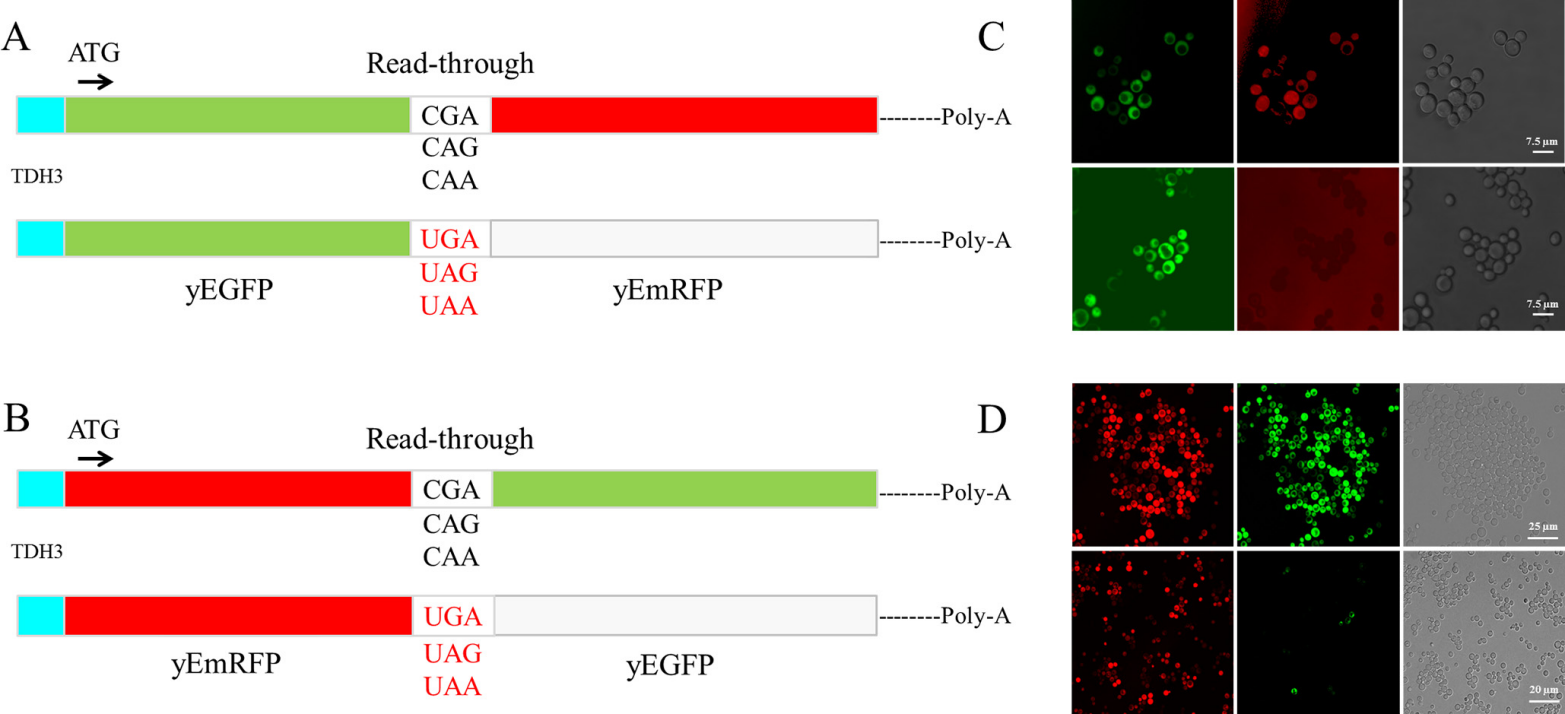
Read-through reporter systems. (A) Plasmids of the YEpGR series harboring the yEGFP and yEmRFP coding sequences separated by either an in frame sense or nonsense codon; (B) Constructs bearing the same yEmRFP and yEGFP ORFs cloned in the inverted order; (C) Fluorescence microscope images of yeast cells transformed with plasmids expressing a yEGFP-sense-yEmRFP construct (CGA) or yEmRFP-nonsense-yEGFP (UGA) configuration; (D) plasmids expressing a yEmRFP-sense-yEGFP (CGA) or yEmRFP-nonsense-yEGFP (UGA) read-through cassette.

**Fig 2:**
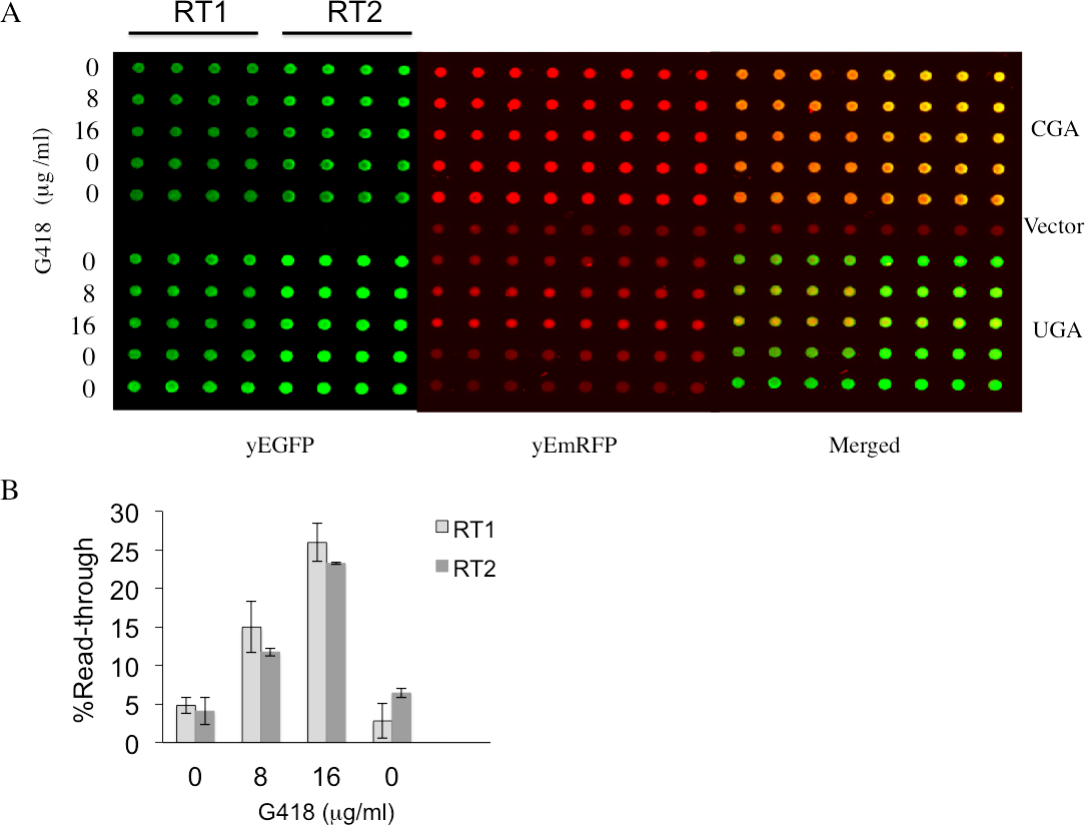
Read-through mediated by G418 determined by using a dual-laser fluorescence scanner. (A) Yeast strain CW04 was transformed with plasmids (YEpGR series) harboring the indicated read-through cassette UGA or the corresponding sense control CGA. Independent clones were inoculated in quadruplicates in 96 wells microplates to perform two read-through assays (RT1 and RT2). Geneticin (G418) was added at the concentrations indicated. Microplates OD was measured at 595 nm and fluorescence was acquired by using the dual-laser scanner Typhoon 8600 after 24h incubation at 30°C. (B) Read-through levels as a function of the presence of increasing concentrations of G418 were quantitate as described in the Materials and Methods. Quantitative data were obtained from two independent experiments and are expressed as mean values and indicated with standard deviation.

**Fig 3:**
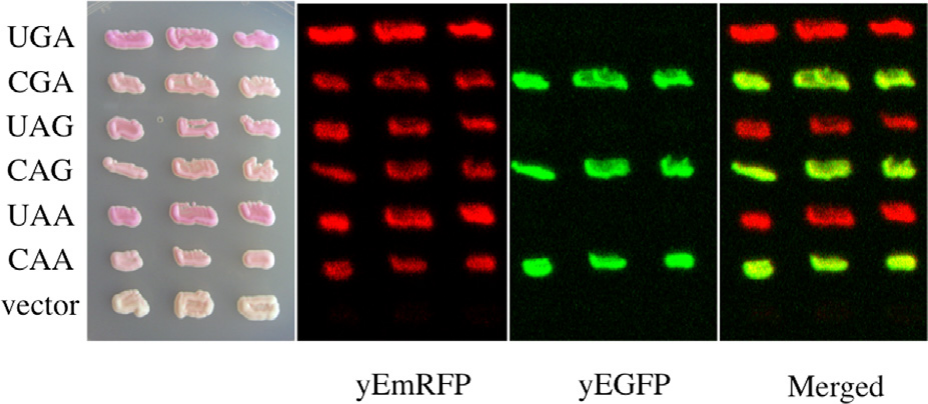
Expression of YEpRG reporters in yeast. Yeast cells were transformed with plasmids of the YEpRG series based on the constructs depicted (Fig 1B) which are expressing the read-through cassettes indicated. Yeast cells transformed with a YEpRG expressing the yEmRFP and yEGFP ORFs with an in frame sense codon (CGA, CAG or CAA) display both the red and green fluorescence, whereas only red fluorescence could be expressed from those plasmids into which a nonsense codon (UGA, UAG or UAA) was placed between the yEmRFP and yEGFP ORFs. Yeast transformants were selectively grown on solid synthetic minimal medium in the absence of uracil. Imaging was performed by using the dual laser scanner and acquired after overnight incubation of the plate at 30°C.

Most read-through assays at PTCs are currently performed by using reporter systems based on luciferases. Particularly, in the dual luciferase system, Renilla and firefly encoding sequences are cloned in tandem and placed in phase such as to express a unique open reading frame or the two sequences are separated by a stop codon [25–28]. In the latter configuration the sequence of firefly downstream to the stop codon can be expressed only if read-through does occur thus providing a quantitative measure of read-through at the stop codon separating the two sequences. Taking advantage of this robust principle a rapid and efficient read-through reporter system based on dual fluorescence based on the yeast *Saccharomyces cerevisiae*, for which genetic tools are available was developed [26]. To construct a dual-fluorescence reporter system the sequences encoding the yEGFP (yeast-enhanced green fluorescent protein) and a variant of the mCherry red fluorescent protein (RFP) were used. The latter RFP variant sequence was codon-optimized for the expression in *Saccharomyces cerevisiae* as yeast-enhanced mRFP (yEmRFP) and can combine fluorescence and a purple visible phenotype [29]. A powerful color phenotype read-through assay was successful developed in yeast [30, 31]. The feasibility in using the two fluorescent proteins as read-through reporters, whose expression is easily detectable *in vivo*, was then explored. The YEpGAP expression plasmid bearing the yEmRFP constructed by N. Dean and colleagues was used [29]. This plasmid constitutively expresses yEmRFP under the control of the *TDH3* promoter and confers a characteristic pink color to yeast transformants. The yEGFP upstream to yEmRFP was cloned and the intergenic sequences subsequently modified by site-directed mutagenesis to separate the two open reading frames (ORFs) by a stop codon UGA, UAG or UAA, or to connect them by a correspondent sense codon CGA, CAG or CAA as depicted (Fig 1A) (see also Materials and Methods). These constructs were then transformed in yeast cells and observed using fluorescence microscopy for fluorescent proteins expression. Yeast cells harboring the YepGR-CGA displayed both the green and red fluorescence (Fig 1C, top), whereas those carrying the YepGR-UGA lack the red fluorescence (Fig 1C, bottom). In order to evaluate the response of our reporter system read-through mediated by the aminoglycoside G418 (geneticin) was determined. Yeast transformants carrying the YepGR-CGA or YepGR-UGA or the vector only, were grown directly in a 96 well microplate in the absence or presence of increasing concentrations of G418 (8 and 16 μg/ml) proved to be efficient in inducing read-through in yeast [32]. After overnight incubation at 30°C the expression of green and red fluorescence were monitored using a dual-laser scanner. Cells harboring YepGR-UGA, that is expressing yEGFP only, clearly showed an increasing red fluorescence as a function of the increasing concentration of G418 thus rendering visible the phenomenon of read-through (Fig 2A). Fluorescence in each well was quantified by using IQTL software and read-through percentage was calculated as described (Fig 2) see also Materials and Methods and the Supporting Information (S1 Fig). The ability of the aminoglycoside G418 in mediating read-through in a dose dependent manner was clearly shown in two parallel read-through assays with independent clones (RT1 and RT2) (Fig 2B). Following these results read-through was tested at the stop codons UAG and UAA in yeast transformants bearing YepGR-UAG, YepGR-CAG, YepGR-UAA or YepGR-CAA. Difficulty was found in measuring read-through at these stop codons. The UAG and UAA stop codons are known to be less susceptible to read-through than UGA and it is likely that the background of natural red fluorescence, at least in our yeast strain, constitute an important interference (results not shown). In order to circumvent this shortcoming a novel plasmid set was constructed into which the order of the fluorescent proteins was inverted as shown in Fig 1B. The novel set of plasmids (YepRG series) was transformed into yeast and transformants were checked for the correct expression of the dual red-green fluorescence. In Fig 1D yeast cells harboring either YepRG-CGA or YepRG-UGA expressing both red and green fluorescent proteins or only the red protein are shown. A similar expression pattern was observed from fluorescence microscopy for the other sense and nonsense codons (not shown). The potential visible color phenotype in our strain was correlated to fluorescence. Novel yeast transformants were streaked as a patch on a selective solid medium and grown overnight at 30°C. Imaging of the relevant plate revealed a pink color displayed by all transformants, more intense in the transformants bearing the YepRG-UGA, YepRG-UAG or YepRG-UAA plasmids expressing only yEmRFP and absent in the control vector (Fig 3). Only transformants bearing the sense codons expressed the green fluorescent protein and no green background was observed in cells expressing the plasmids bearing a stop codon between the two reporter sequences.

### Dual fluorescence reporter system responds to both G418 and Gentamicin at all stop codons

**Fig 4:**
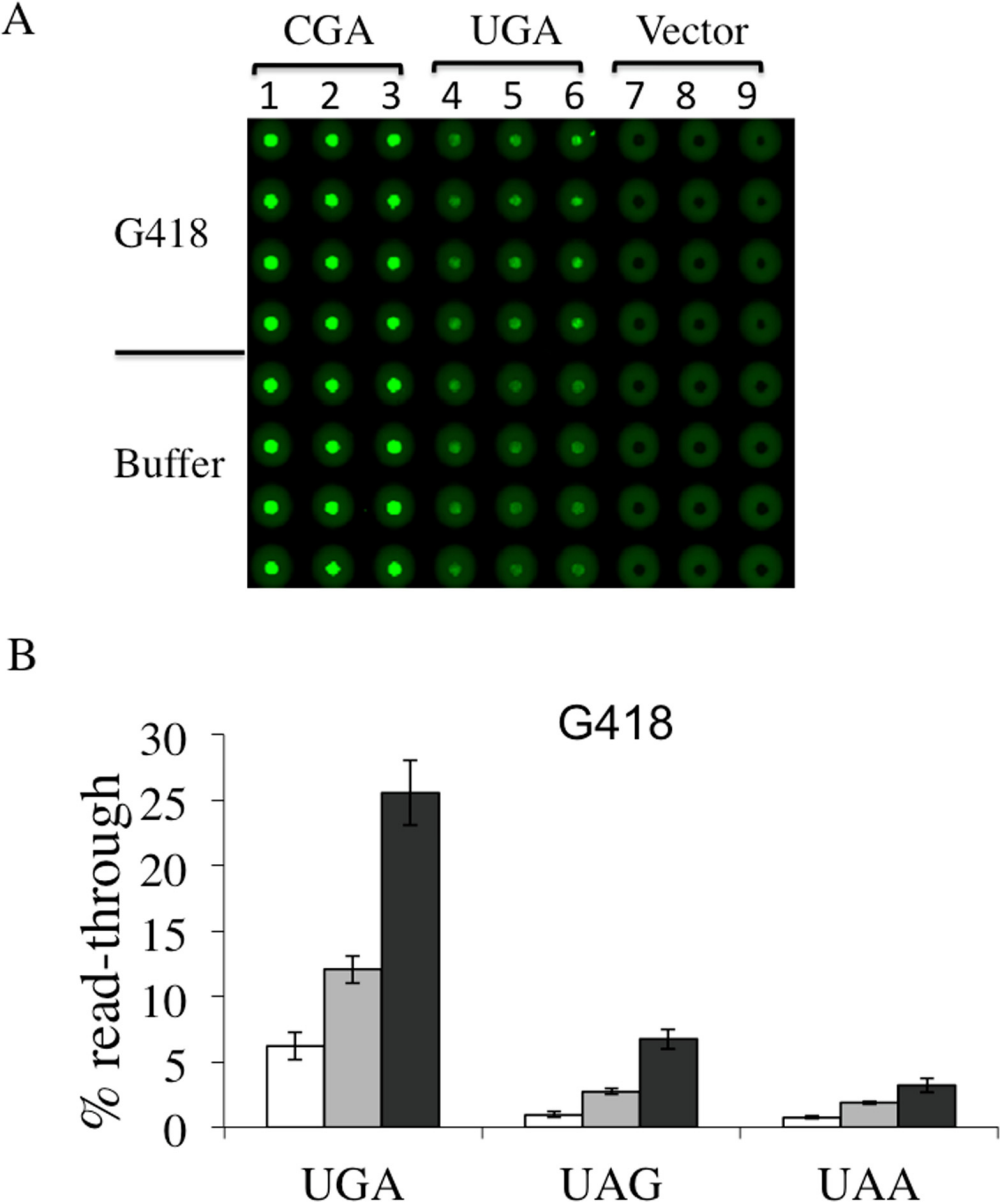
Read-through efficiency at UGA, UAG and UAA premature stop codons mediated by aminoglycoside G418 determined by YEpRG dual fluorescent reporters. Yeast transformants harboring the YEpRG series (Figs 1B and 3), bearing each UGA, UAG or UAA premature stop codon, or the corresponding sense codon controls, inserted between the yEmRFP and yEGFP ORFs were grown in liquid selective medium and inoculated in quadruplicate in 96 wells microplates in the absence or presence of G418 (8–16 μg/ml). Dual fluorescence was acquired as in Fig 2 (see also text). A) Shown is a representative image of yEGFP acquired by a Typhoon 9600 FLA after 19h incubation at 30°C related to a G418 mediated read-through assay at the UGA stop codon. G418 was added at 8 μg/ml (lanes 2, 5 and 8) or 16 μg/ml (lanes 3, 6 and 9) (primary data and experimental scheme are available in the Supporting Information (S2 Fig). B) Read-through percentage is calculated as described in the Materials and Methods. Data are expressed as mean values and indicated with standard deviation.

**Fig 5:**
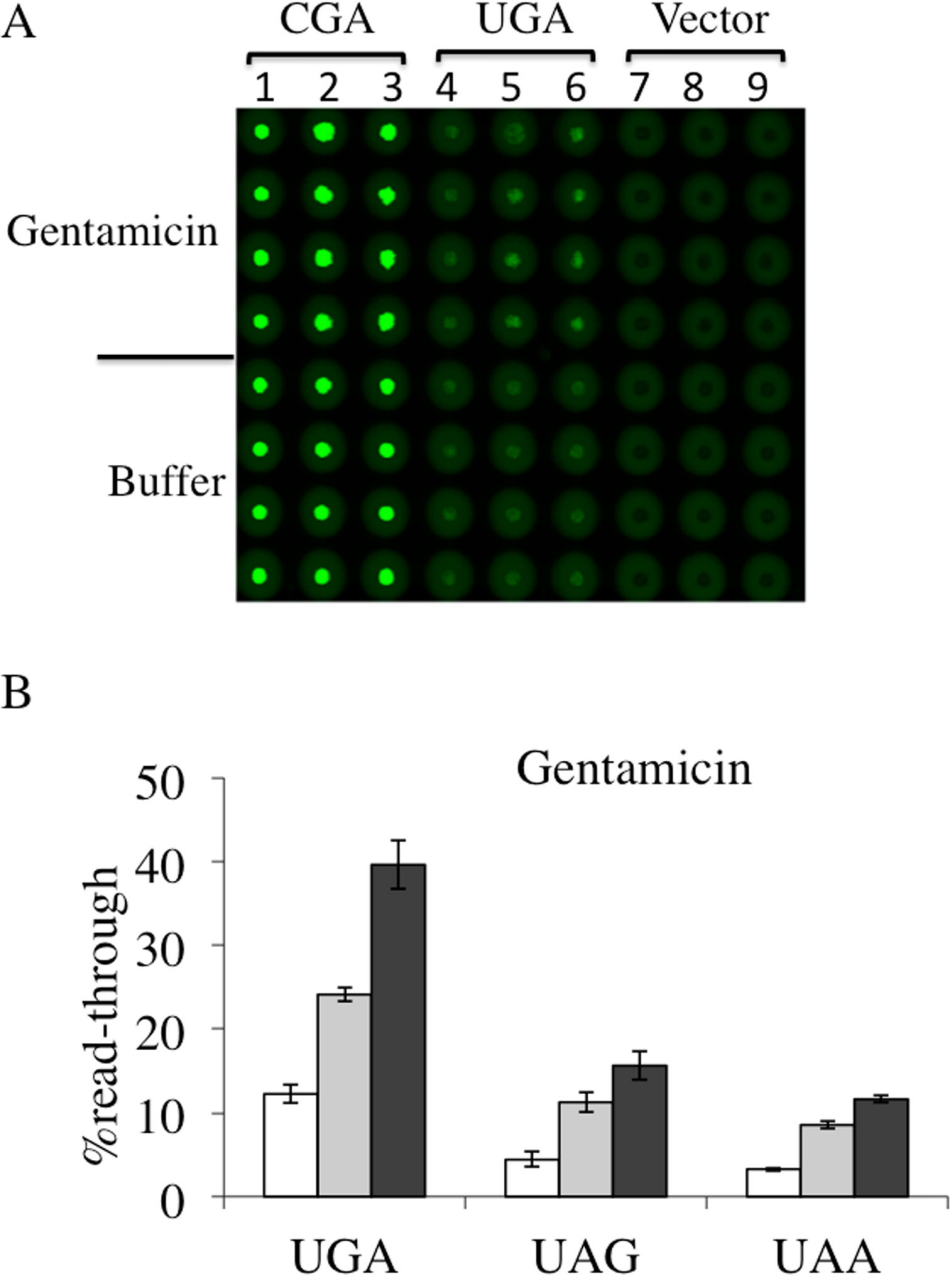
Read-trough efficiency at UGA, UAG and UAA premature stop codons mediated by Gentamicin. Yeast transformants with a plasmid harboring each of the nonsense or the correspondent sense codon, were prepared and cultivated in quadruplicates as described (Fig 4), in the absence or presence of aminoglycoside gentamicin, added at 200 μg/ml (lanes 2, 5 and 8) or 400 μg/ml (lanes 3, 6 and 9. A) Shown is a representative image of yEGFP acquired by a Typhoon 9600 FLA after 19h incubation at 30°C related to a gentamicin mediated read-through assay at the UGA stop codon (see also S2 Fig). B) Read-through percentage is calculated as described in the Materials and Methods. Data are expressed as mean values and indicated with standard deviation.

In order to evaluate the dual fluorescence reporter system a comparative assay was conducted with compounds well characterized for their efficiency in inducing read-through at premature termination codons. Aminoglycosides G418 (geneticin) and gentamicin are validated read-through correctors in different experimental cell systems and demonstrated to possess the ability to restore full-length production of the cystic fibrosis transmembrane conductance regulator (CFTR) in a bronchial cell line carrying a nonsense mutation in the CFTR gene [33], a functional full-length p53 protein in human cancer cell line containing a PTC [34] and the biological activity of the adenomatous polyposis coli PTC-gene in human cancer cells [35]. In a previous study G418 and gentamicin were shown to be efficient in inducing dose-dependent read-through at stop codons by using a dual luciferase reporter expressed in yeast [32]. Read-through assay was performed using the dual fluorescence reporters, to monitor and quantify the effects of both aminoglycosides at all stop codons. Yeast transformants with YEpRG series plasmid reporters each carrying UGA, UAG or UAA read-through cassette as well as the correspondent sense controls CGA, CAG or CAA transformants were incubated with G418 or with gentamicin at the same concentrations previously used, 8 and16 μg/ml, and 200 and 400 μg/ml, respectively. In panels A of Figs 4 and 5 we report the results obtained using Microplates incubated overnight at 30°C and scanned for dual fluorescence after 19-24h by a Typhoon 9600 FLA (GE Healthcare). yEGFP was revealed at 478 excitation and 532 nm emission, whereas yEmRFP at 540 excitation and 635 emission. The quantitative data relative to the obtained fluorescence was acquired by the IQTL software (GE Healthcare) and exported as Excel files that are available in the Supporting Information (S2 Fig). Read-through percentage was calculated as described in the Material and Methods section and reported in panels B of Figs 4 and 5. Read-through was expressed as the ratio Green/RED (nonsense) divided by the ratio Green/RED (sense) x 100. The percent read-through is expressed as the mean ± the standard deviation. All reported values are obtained by at least three independent experiments. The results obtained clearly show that both G418 (Fig 4) and gentamicin (Fig 5) exhibited efficient read-through capability, after 19-24h of incubation, at all three stop codons. These results reiterate our previous findings about the read-through properties of aminoglycosides characterized by using the yeast based dual-luciferase assay [35], indicating that this novel dual fluorescence read-through reporter system is at least as powerful as the dual luciferase based assay. Taken together, these results indicate that this novel dual-fluorescence reporter system based on yeast is sensitive to both widely characterized read-through molecules and is suitable for a general primary screening at all three premature stop codons.

### Dual fluorescence reporter system responds to NMD

**Fig 6:**
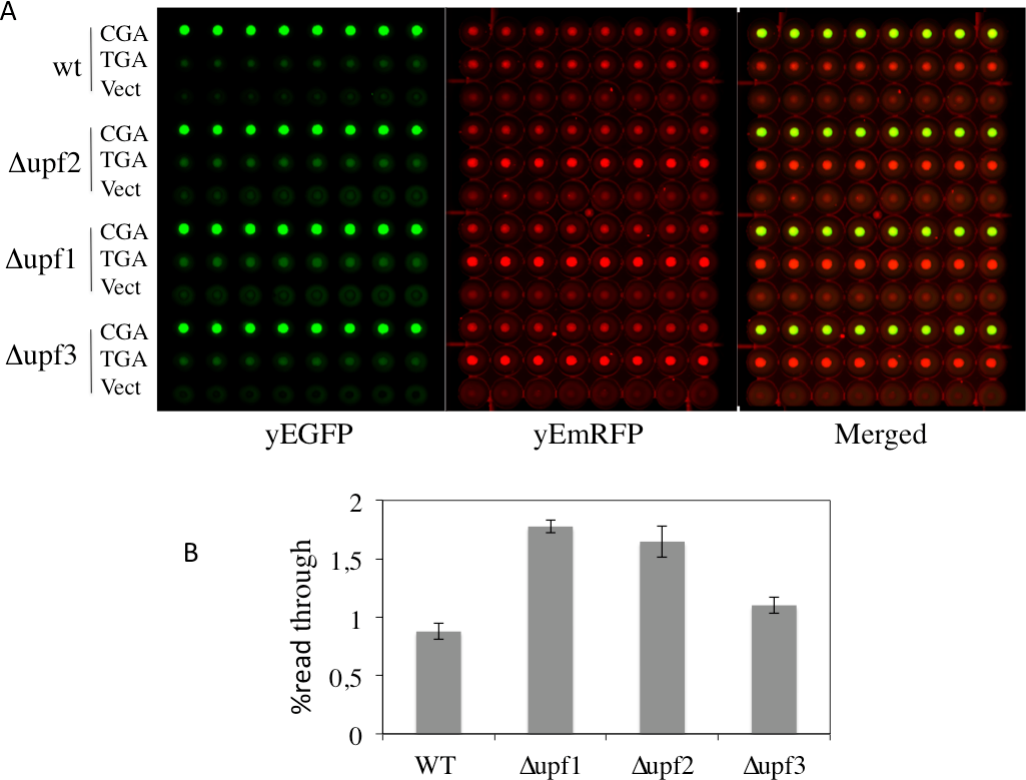
Read-through assay as a function of abolished NMD. WT and Δ*upf1*, Δ*upf2* and Δ*upf3* strains were transformed with the YepRG-UGA or YepRG-CGA reporters. A) Yeast transformants were inoculated incubated at 30°C for 24 h in microplate and analysed by the dual fluorescence scanner (Typhoon FLA9600) as described in the previous experiments. B) Read-through percentage was calculated as described in the Materials and Methods. Data are related to identical clones replicates and expressed as mean values and indicated with standard deviation. Wild type and upfs deleted strains are described in Materials and Methods.

**Fig 7:**
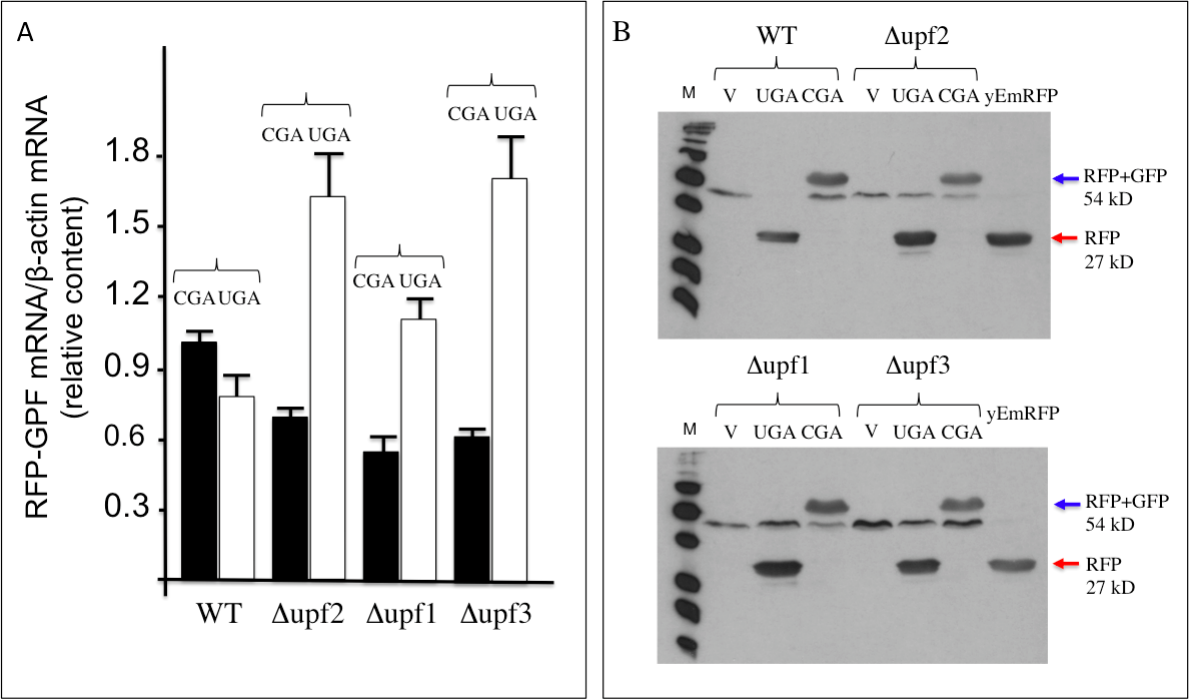
Expression products of the YepRG-UGA and YepRG-CGA reporters in the wild type and Δ*upf1*, Δ*upf2* and Δ*upf3* strains. (A) Quantification (RT-qPCR) of full length yEmRFP-yEGFP mRNAs in WT and Δ*upf1*, Δ*upf2* and Δ*upf3* mutants transformed with YepRG-UGA or YepRG-CGA. The relative values (RFP-GFP mRNA/β-actin) are indicated with black boxes (UGA clones) and white boxes (CGA clones). (B) Western blotting with cellular extracts obtained from WT and upfs mutants transformants. Western blotting was performed using an antibody against RFP protein, detecting the full-length yEmRFP-yEGFP (blue arrowed) as well as truncated yEmRFP-stop (red arrowed) proteins. Yeast anti-Actin antibody was used for densitometry normalization (data not shown). UGA, clones containing the read-through UGA cassette; CGA, clones containing the sense control CGA cassette; V, clones containing the empty vector; M, markers.

**Fig 8:**
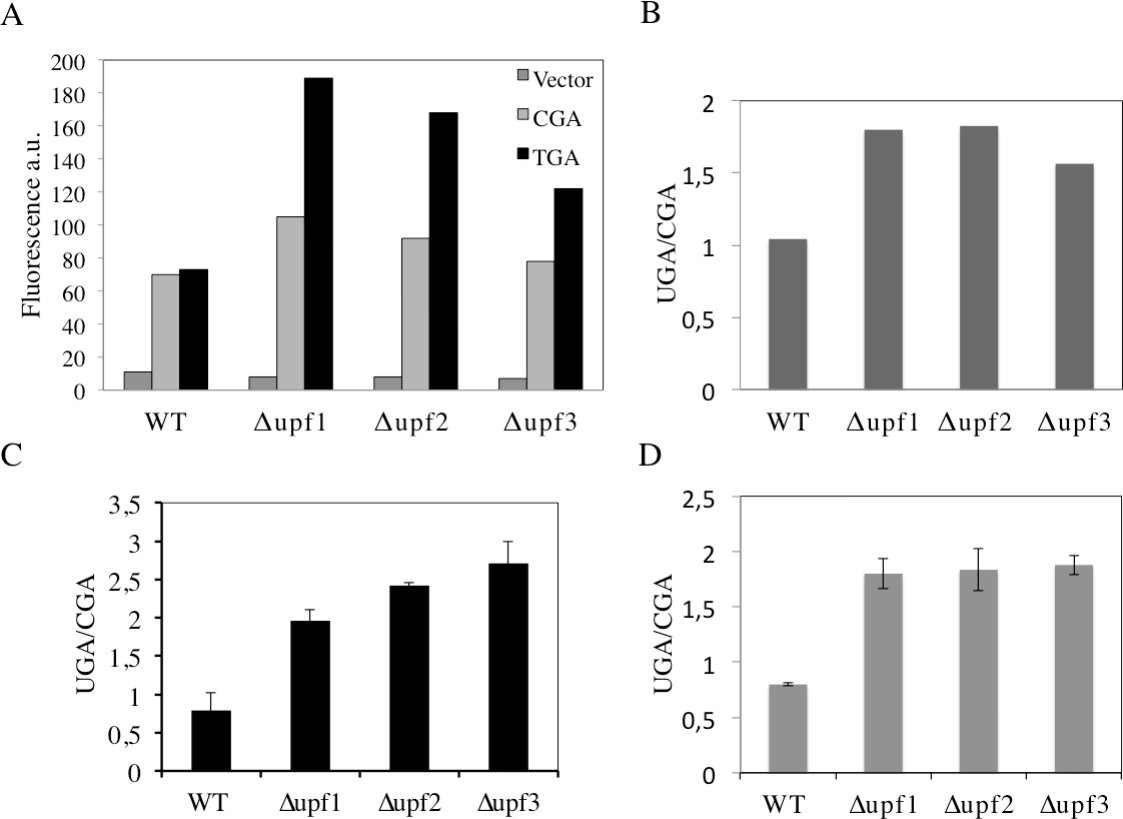
Dual fluorescence reporter response to NMD is recapitulated in flow cytometry analysis. A) WT and Δ*upf1*, Δ*upf2* and Δ*upf3* strains harboring the YepRG-UGA or YepRG-CGA reporters were analyzed by a flow cytometer. yEmRFP fluorescence was measured by observing 10.000 yeast cells as described in Materials and Methods. B) UGA/CGA fluorescence ratio derived from data in A; C) UGA/CGA ratio calculated from RT-qPCR analysis (Fig 7A); D) UGA/CGA ratio calculated from microplate assay (Fig 6A). Data are expressed as mean values and indicated with standard deviation.

It is well established that the core system of NMD is conserved from yeast to human and its function depends on the *UPF1*, *UPF2* and *UPF3* genes [36]. In yeast deletion of each of these genes abolishes NMD with a consequent increase in the abundance of PTC-containing mRNAs and, in addition, results in a nonsense suppression phenotype (read-through) [27, 37, 38]. In order to verify that a stop codon inserted in frame between the yEmRFP and yEGFP sequences in our reporters is actually recognized as a PTC and triggers NMD, we have tested our dual fluorescence reporter system in strains into which NMD was either functional or abolished by deletion of the *UPF1*, *UPF2* or *UPF3* genes. To this aim, wild type (WT) and Δ*upf1*, Δ*upf2* or Δ*upf3* deleted strains were transformed with the YepRG-UGA or YepRG-CGA reporter plasmids and a read through assay was performed. Results shown in Fig 6A display both the yEmRFP and yEGFP fluorescence acquired by the dual fluorescence scanning as previously described in the drugs mediated read though assay (Figs 4 and 5). It can be noted that the red fluorescence related to the samples expressing the yEmRFP-UGA-yEGFP reporter was significantly increased in the Δ*upf1*, Δ*upf2* or Δ*upf3* strain with respect to WT. In addition, an increase in the green fluorescence can be appreciated in the upfs deleted strains compared to WT. Consistently, an increase of read through in the Δ*upf1*, Δ*upf2* or Δ*upf3* strain (up to 2 folds) with respect to wild type was observed and quantified in Fig 6B. Thus, our read-though reporter system appears to be able to sense the NMD functional state and the nonsense suppression phenotype associated with lack of NMD. In order to verify mRNA stability and translation products in those genetic contexts, we next examined the abundance of both the yEmRFP-UGA-yEGFP and yEmRFP-CGA-yEGFP transcripts and translation products expressed by the corresponding YepRG-UGA and YepRG-CGA reporter plasmids. In the experiments depicted in Fig 7 RNA transcription (Fig 7A) and full-length yEmRFP-yEGFP protein production (Fig 7B) was detectable in all the YepRG-CGA clones (either WT, or Δ*upf1*, Δ*upf2* and Δ*upf3*). As expected, in all the YepRG-UGA clones, only the truncated yEmRFP-UGA-yEGFP protein was detected (Fig 7B). Quantitative analysis on RT-qPCR results (Fig 7A) showed that in all the YepRG-UGA clones the relative content of yEmRFP-UGA-yEGFP mRNA is higher when Δ*upf1*, Δ*upf2* and Δ*upf3* are compared to the WT, supporting the hypothesis that, in the absence of NMD, mRNA with the UGA in frame is more stable. This is well in agreement with the Western blotting analysis, showing in these YepRG-UGA transformants a higher content of protein products in Δ*upf1*, Δ*upf2* and Δ*upf3* than the WT (normalized yEmRFP densitometric values were as follows: WT 10%; Δ*upf2* 14%; Δ*upf1* 22%; Δ*upf3* 12%). In these samples we were unable to detect the full-length protein yEmRFP-yEGFP that would result from read through at the UGA codon, probably because the experimental conditions used were below the detection limit. In addition, slight differences were observed likely depending on intrinsic variability due to the experimental manipulations performed to generate the Δ*upf1* samples (Fig 7). In this context, we sought to further check the system by using a different technique such as flow cytometry to measure yEmRFP fluorescence at the cellular level and estimate the mRNA abundance reference ratio UGA/CGA. As expected, the yEmRFP fluorescence increased in Δ*upf1*, Δ*upf2* and Δ*upf3* bearing the YepRG-UGA reporter compared to WT and with respect to transformants harboring the YepRG-CGA reporter (Fig 8A). Comparison of the UGA/CGA ratio of yEmRFP fluorescence resulting from flow cytometer (Fig 8B) with that obtained from RT-qPCR analysis (Fig 8C) and microplate scanning (Fig 8D) showed a very similar trend of the increase profile.

Taken together, these results demonstrated that the dual fluorescence reporter system responds to NMD and nonsense suppression associated to the absence of NMD.

## Discussion

Recent progress in the chemical-induced read-through approach to overcome PTCs has led to the identification of several compounds possessing read-through ability and of potential therapeutic application in several pathologies caused by nonsense mutations. This property has been screened and examined throughout several cell-culture models of different diseases and a number of different methods [2, 9]. However, the read-through response to chemicals is highly variable and many studies indicate that various factors play a role in the efficacy of read-through. Basal suppression of stop codons UGA (opal), UAG (amber) or UAA (ochre), is known to display an UGA >UAG >UAA hierarchy in the read-through efficiency and is strongly influenced by the identity of the nucleotide immediately downstream to stop codons (position +4). The read-through profile can also be altered when induced by drugs [8, 24]. Studies in the yeast *Saccharomyces cerevisiae*, in which eukaryotic translation termination is conserved, have highlighted the rules governing translation termination efficiency. The simple eukaryotic model was the first genetic context into which suppression of nonsense mutations mediated by paromomycin, an aminoglycoside, was shown [30, 31]. In this study a yeast-based *in vivo* enzyme-independent method that is rapid, inexpensive, sensitive, quantitative and suitable for the screening of low molecular weight compounds able to promote read-through at PTCs has been developed. This method is based on the use of a set of reporter plasmids harboring the coding sequences of two fluorescence proteins, yEmRFP and yEGFP, both being adapted to be expressed in the yeast *Saccharomyces cerevisiae* and endowed with read-through cassettes separating the two open reading frames. GFP-reporter cell-based assay for translational read-through have been recently described, with a stop codon introduced either within the ORF of interest [39] or inside the GFP reporter ORF [40]. The dual fluorescence system takes advantage of the expression system of the robust dual luciferase reporter [25, 27], for which the yEmRFP and yEGFP expression is under control of a single constitutive promoter (TDH3). This is transcribed as a unique mRNA and translated by a single ATG start codon as a unique ORF (see Fig 1). Normalization of nonsense to sense reporter expression takes into account effects of drugs on protein synthesis. A similar principle was successfully adopted for programmed -1 ribosomal frame shifting assay in yeast [41]. As a stop codon separating the two ORFs serves as PTC, the relevant mRNA is substrate for NMD and the ORF upstream of the stop codon, i.e. yEmRFP, provides a reference of mRNA abundance (see Figs 7 and 8). The reporters were evaluated for yEmRFP and yEGFP cloning order. The study showed that yEGFP placed downstream of the PTC, while exhibiting read-through efficiency equivalent for the UGA stop codon in the inverted order is most effective for both the UAG and UAA stop codons in absence of natural green fluorescence in our yeast strain. Combining multiwells format with a dual-laser fluorescence scanning allows rapid data acquisition and export in excel spreadsheets with results obtained in less than 24 h. Normalization of data with yeast cells OD595, provides monitoring of eventual toxic effects of tested compounds. The method has been evaluated with G418 and gentamicin, the most extensively studied aminoglycosides for the read-through approach with G418 being more efficient in read-through [2]. G418 promotes read-through at a comparable level but at concentrations 25-fold less than gentamicin (Figs 4 and 5). The read-through profile at each stop codon is in general agreement with those previously reported, as determined by dual luciferase reporters in yeast [32]. This novel read-through reporter system responds to aminoglycosides mediated read-through working equally well at all stop codons.

In the present study a yeast-based *in vivo* dual fluorescence read-through system that proved to be extremely simple and robust in the screening for the effect of drugs at each stop codon UGA, UAG and UAA in the same nucleotide context was tested. Results show that the read-through response to low molecular weight drugs in yeast is essentially consistent with recent findings in human cells where enzyme-reporters are mostly used and the simple eukaryote could constitute an invaluable tool in the study of the mechanism of different classes of read-through compounds.

This system should be validated in the next future with the several read-through molecules recently proposed, such as for example NB30, NB54, NB84 [14, 42, 43] and possibly other nonaminoglicoside compounds including Ataluren (PTC124) [19], RTC13 and RTC14 [20] GJ071and GJ072 [21] and Amlexanox [22]. In addition the results obtained with this *Saccharomyces cerevisiae* based method should be comparatively assessed with *in vitro* experimental systems based on eukaryotic cell lines harboring target genes carrying premature termination codons to be suitably corrected with read-through inducing molecules [9].

Fast high throughput screening of drugs of possible interest in experimental therapy of diseases caused by nonsense mutations is of great interest in medicinal chemistry, given the high number of patients affected by these disorders. The screening of new molecules and characterization of drugs already used in therapy or clinical trials for other pathologies could provide a repurposing/repositioning strategy for drugs to treat diseases caused by nonsense mutations [44]

## Materials and Methods

### Yeast strains and plasmids

The yeast *S*. *cerevisiae* strain used in the present study is CW04, a derivative of W303-1A (*MATa*, *his3–11*, *his3–15*, *leu2–3*, *leu2–112*, *ade2–1*, *ura3–1*, *trp1–1*, *can1–100*). Strains deleted of Upf1 (HFY871), Upf2/Nmd2 (HFY1300) or Upf3 (HFY861) and the corresponding wild type (HFY114) were described in [38, 45, 46]. Yeast cells were cultivated and transformed using the LiCl procedure as described [32].

### Construction of the YEpGR serie reporters

The structure of dual fluorescence reporter is based on the original plasmid yEpGAP-Cherry, a high copy 2μ, URA3 vector, carrying the yEmRFP sequence. whose expression is placed under the control of the constitutive promoter TDH3 [29]. YepGAP-Cherry was first used as a template in site-directed mutagenesis PCR to substitute the ATG start codon in yEmRFP. This can be with either the CGA sense codon or the TGA stop codon. A Bgl II restriction site can be introduced upstream of the codon by two site-directed PCRs using the forward primers 3 or 4 respectively and the reverse primer 5 introducing a Xba I site (Table 1).

**Table 1:** List of primers.

1	yEGFP-Fw	GAGCTCAGATCTGCTCGTCGACCTCGACATGTCTA
2	yEGFP-Rv	GCAGGATCTAGACATTTTGTACAATTCATCCATAC
3	GR-CGA-Fw	ATGTCTAGATCCTGCGCACGACCCGGGGTTTCAAAAGGTGAAGAAG
4	GR-TGA-Fw	ATGTCTAGATCCTGCGCATGACCCGGGGTTTCAAAAGGTGAAGAAG
5	GR-Rv	AGCAGATCTGAGCTCTTTGTTTGTTTATGTGTGTTTATTCG
6	GR-Fw-CAG	CAGCCCGGGGTTTCAAAAGGTGAAG
7	GR-Fw-TAG	TAGCCCGGGGTTTCAAAAGGTGAAG
8	GR-Fw-CAA	CAACCCGGGGTTTCAAAAGGTGAAG
9	GR-Fw-TAA	TAACCCGGGGTTTCAAAAGGTGAAG
10	Ch Fw-EcoRI	CTGACTGAATTCATGGTTTCAAAAGGTGAAGAAGA
11	Ch-Rev-Sal I	CTGACTGTCGACTTTATATAATTCATCCATACCACCAG
12	yEGFP-Rv-XhoI	ATCACGTCTCGAGGGCGTGAATGTAAGCGTGAC
13	RG-Fw-CGA	CGACCCTCTAAAGGTGAAGAATTATTCACTGGTGTTG
14	RG-Fw-TGA	TGACCCTCTAAAGGTGAAGAATTATTCACTGGTGTTG
15	RG-Fw-CAG	CAGCCCTCTAAAGGTGAAGAATTATTCACTGGTGTTG
16	RG-Fw-TAG	TAGCCCTCTAAAGGTGAAGAATTATTCACTGGTGTTG
17	RG-Fw-CAA	CAACCCTCTAAAGGTGAAGAATTATTCACTGGTGTTG
18	RG-Fw-TAA	TAACCCTCTAAAGGTGAAGAATTATTCACTGGTGTTG
19	YEpRG-Rv	GGTACCTTTATATAATTCATCCATACCACCAG

Restriction sites introduced with primers are underlined

The yeast-enhanced green fluorescent protein (yEGFP) sequence was amplified by PCR, using the plasmid pUG35 as a template (U. Güldener and J. Hegemann, unpublished results), with primers 1 and 2 (Table 1) and cloned in the Bgl II and Xba I restriction sites to form the recombinant YEpGR-CGA and YEpGR-TGA plasmids. YEpGR-CGA was then used as a template in site-directed mutagenesis PCRs to convert the sense codon CGA into the nonsense UGA or UAA codon. YEpGR-TGA was used as a template to convert the nonsense codon TGA into the sense CGA or CAA codon.

### Construction of the YEpRG serie reporters

The plasmid YepGAP-Cherry [29] was used as a template to amplify the yEmRFP coding sequence, with primers 10 and 11, to clone the sequence as an Eco RI/Sal I amplicon into the polylinker of the plasmid pUG35, upstream of yEGFP. The yEmRFP-yEGFP fusion obtained was amplified using the primers 10 and 12 and cloned back into the EcoRI-XhoI restriction sites of the YEpGAP vector to obtain the recombinant YEpRG plasmid. This was used as a template to introduce the read-through cassettes in frame between the yEmRFP and yEGFP ORFs by 6 PCRs using each forward primer 13–18 with the Rv primer 19.

Site-directed mutagenesis PCRs were performed by using Phusion Hot Start II High-Fidelity DNA Polymerase (Thermo Scientific) or Q5 Hot Start High-Fidelity DNA Polymerase (New England Biolabs Inc.) according to the supplier instructions. Corrected mutagenesis issues were all verified by sequencing.

### Read-through assay

Yeast transformants bearing a plasmid with a nonsense cassette, or the correspondent sense control or vector alone were loaded on 96 wells microplates as follows. Yeast transformants cultures were grown overnight at 30°C in selective medium without uracil, diluted at OD595 = 0.00125 and incubated 4 h before being dispensed as 150 μl per well in quadruplicate. Chemicals to be tested were added at the concentrations indicated. Sample controls in the absence of chemicals were compensated with an equal volume of water (G418 and geneticin). Microplates were incubated overnight at 30°C, then read for OD595 and scanned for dual fluorescence after 16-24h by a Typhoon 8600 (Amersham) or Typhoon 9600 FLA (GE Healthcare). yEGFP was revealed at 478 excitation and 532 nm emission, whereas yEmRFP at 540 excitation and 635 emission. Fluorescence for quantitative data was acquired by IQTL software (GE Healthcare) and exported as Excel files. Read-through percentage was calculated as follows:

For YEpGR transformants, fluorescence was first normalized to OD values and subtracted of vector transformed samples, then read-through was expressed as the ratio RED/Green (nonsense) divided by the ratio RED/Green (sense) x 100;

For YEpRG transformants the same procedure was followed unless read-through was expressed as the ratio Green/RED (nonsense) divided by the ratio Green/RED (sense) x 100.

The percent read-through is expressed as the mean ± the standard deviation. All reported values are obtained by at least three independent experiments. Independent clones (indicated for instance as RT1 and RT2 in Fig 2) were usually inoculated in quadruplicates in the 96 wells microplates, in order to obtain data about intra-plate variation and statistical significance of the average data obtained. Where indicated identical clones replicates were inoculated as indicated in Fig 6.

### Protein extracts preparation

Yeast cells pellets were resuspended in an appropriate amount of Y-PER Yeast Protein Extraction Reagent (Thermo Scientific) added of 100X Halt Protease Inhibitor Cocktail (Thermo Scientific). The amount of Y-PER Yeast Protein Extraction Reagent was calculated according to the weight of pellet as indicated the manufacturer’s instructions. The mixtures ware agitated at room temperature for 20 minutes and then, centrifuged at 14,000g for 10 minutes at 4°C. The supernatant that contains proteins was reserved and stored at -80°C. Protein extracts were quantified using Pierce BCA Protein Assay Kit (Thermo Scientific).

### Western blotting

65 μg of cytoplasmic extracts were denatured for 5 min at 98°C in 1x SDS sample buffer (62.5 mM Tris-HCl pH 6.8, 2% SDS, 50 mM Dithiotreithol (DTT), 0.01% bromophenol blue, 10% glicerol) and loaded on SDS-PAGE gel (10 cmx8 cm) in Tris-glycine Buffer (25 mM Tris, 192 mM glycine, 0.1% SDS). Precision Plus Protein WesternC Protein Standards (size range of 10–250 kDa) (BioRad) was used as standard to determine molecular weight. The electrotransfer to 20 microns nitrocellulose membrane was performed using Trans-Blot Turbo Transfer System (BioRad) with Turbo program (2.5A, 25V, 7 minutes). The membranes were prestained in Ponceau S Solution (Sigma Aldrich) to verify the transfer, washed twice with 25 ml TBS (10 mM Tris-HCl pH 7.4, 150 mM NaCl) for 5 minutes at room temperature and incubated in 30 ml of blocking buffer for 1 hour at room temperature. The membranes were washed three times for 5 minutes each with 30 ml of TBS-T (TBS, 0.1% Tween-20) and incubated with Anti-RFP primary mouse monoclonal antibody (1:2000) (Cat. AKR-021, Cell Biolabs, San Diego, CA, USA) in 10 ml primary antibody dilution buffer with gentle agitation over-night at 4°C. The day after, the membranes were washed three times for 5 minutes each with 30 ml of TBS-T and incubated in 10 ml of blocking buffer, in gentle agitation for 1 hour at room temperature, with Stabilized Goat Anti-Mouse IgG, (H+L), Peroxidase Coniugated secondary antibody (1:2000)(Thermo Scientific) and Precision Protein Strep-Tactin HRP-conjugated antibody (1:10.000) (BioRad) used to the protein marker. Finally, after three washes each with 30 ml of TBS-T for 5 minutes, the membranes were incubated with 5 ml LumiGLO^®^ (0.5 ml 20x LumiGLO^®^, 0.5 ml 20x Peroxide and 9.0 ml Milli-Q water) (Cell Signaling) in gentle agitation for 5 minutes at room temperature and exposed to x-ray film (Pierce, Thermo Scientific). After stripping procedure using the Restore™ Western Blot Stripping Buffer (Pierce) membranes were incubed again in blocking buffer and then reprobed with Anti-Actin primary mouse monoclonal antibody (1:500)(Cat.69100, ImmunO, MPBiomedicals, LLC, Solon, Ohio, USA) used as normalization control. X-ray films for chemiluminescent blots were analyze by Gel Doc 2000 (Bio-Rad Laboratoires, MI, Italy) using Quantity One software to elaborate the intensity data of the target protein.

### RNA extraction

RNA extraction was performed using RiboPure-Yeast Extraction Kit (Ambion) according to the manufacturer’s instructions. Yeast pellet was resuspended 480 μl of Lysis Buffer, 48 μl of 10% SDS and 480 μl of Phenol:chloroform:IAA. The mixture was vortexed vigorously for 15 seconds and then was transferred in a screw cap tube containing 750 μl of Zirconia Beads. Tubes, positioned horizontally, were mixed at maximum speed for 10 minutes and then were centrifuged at 16,000g for 5 minutes at room temperature. The aqueous phase, containing RNA, was transferred in a fresh tube and 350 μl of Binding Buffer was added for each 100 μl of aqueous. 235 μl of 96% DNAse-RNAse free Ethanol was added for each 100 μl of aqueous and the mixture was vortexed. Filter Cartridges were assembled in either Collection Tube and 700 μl of mixture were added. Tubes were centrifuged at 8,000 g for 60 seconds at room temperature and the flow-through was discarded. This step was repeated several times until all the mixture pass through the filter. Filters were washed at first by adding 700 μl of Wash Solution 1, and then by adding twice 500 μl of Wash Solution 2/3. After every wash the filters were centrifuged at 13,000g for 1 minute at room temperature. At the end, the empty filter was centrifuged twice at 13,000g for 60 seconds at room temperature. Filters were transferred to fresh tubes and RNA was eluted with 50 μl Elution Buffer, pre-heated at 95°C. Filters added by Elution Buffer were incubated at room temperature for 5 minutes and then were centrifuged at 16,000g at room temperature at room temperature. To remove DNA contamination, RNA was treated with DNase I. 5 μl of 10X DNase I Buffer and 4 μl of DNase I were added to 50 μl of extracted RNA, and the mixture was incubated for 30 minutes at 37°C.To stop the reaction 5.9 μl of DNase Inactivation Reagent were added to each sample, the solution was vortexed and incubated at room temperature for 5 minutes. The mixture was centrifuged at 16,000 for 3 minutes at room temperature and the supernatant, containing RNA, was transferred in a new tube. RNA obtained stored at -80°C.

### Reverse Transcription and quantitative Real-time PCR (RT-qPCR)

For gene expression analysis 300 ng of RNA treated with DNase I were reverse transcribed to cDNA using SuperScript VILO cDNA Synthesis Kit in a 20-μl reaction. At 300 ng of RNA were added 4 μl of 5X VILO Reaction Mix, 2 μl of 10X SuperScript Enzyme Mix and Rnase-Dnase free water to the final volume of 20 μl. the mixture was incubated for 10 minutes at 25°C, 60 minutes at 42°C and 5 minutes at 85°C. The obtained cDNA was stored at -20°C. Real-time-qPCR experiments were carried using I-Taq Universal SYBR Green Supermix (BioRad), primers (purchased from IDT and Sigma Aldrich) used in the analysis are indicated in the table. cDNA (0,5 μl) was amplified for 45 PCR cycles using an CFX96 Touch Real-Time PCR Detection System (BioRad) in 20 μl final volume reaction mix. Amplification program includes a first denaturation step at 95°C for 3 minutes, followed by 45 cycles consisted of a denaturation phase (96°C for 10 seconds),a phase of primers annealing (63°C for 60 seconds) and finally, an extension phase (72°C for 20 seconds). Relative expression was calculated using the comparative cycle threshold method and the endogenous controls yeast ACTIN, was used as normalizer gene. Duplicate negative controls (no template cDNA) were also run with every experimental plate to assess specificity and to rule out contamination. The real-time PCR reactions were performed in duplicates for both target and normalizer gene.

### Flow cytometry

Yeast cells were grown to OD = 0.5–1. Cell sample was diluted 8 fold with SC buffer and analyzed by using the BD LSRFortessa X-20 (*Becton*, *Dickinson* and Company, Franklin Lakes, NJ, USA) cell analyser. The quartz cuvette flow cell is gel-coupled by refractive index-matching optical gel to the fluorescence objective lens (1.2 NA) for optimal collection efficiency. Emitted light from the gel-coupled cuvette is delivered by fiber optics to the detector arrays. The BD LSRFortessa X-20 uses configurable polygonshaped optical pathways that use signal reflection to maximize signal detection. The flow rate was 12 μL/min and the red emission was recorded in the wavelength range 660–680 nm, in order to avoid any interference effect due to the presence of yEGFP. The number of yeast cells observed in a single analysis was 10,000.

### Materials

Aminoglycosides G418 (A1720) and gentamicin (G1264) were supplied by Sigma-Aldrich.

## Supporting Information

S1 Fig Experimental scheme of dual fluorescence read-through assay by using YEpGR series reporters. (XLSX)

S2 Fig Experimental scheme of dual fluorescence read-through assay by using YEpRG series reporters. (XLSX)

## References

[1] Keeling KM, Xue X, Gunn G, Bedwell DM. Therapeutics based on stop codon readthrough. Annual review of genomics and human genetics. 2014;15:371–94. Epub 2014/04/30. 10.1146/annurev-genom-091212-153527 24773318 PMC5304456

[2] Lee HL, Dougherty JP. Pharmaceutical therapies to recode nonsense mutations in inherited diseases. Pharmacology & therapeutics. 2012;136(2):227–66. Epub 2012/07/24. 22820013 10.1016/j.pharmthera.2012.07.007

[3] Kervestin S, Jacobson A. NMD: a multifaceted response to premature translational termination. Nature reviews Molecular cell biology. 2012;13(11):700–12. Epub 2012/10/18. 10.1038/nrm3454 23072888 PMC3970730

[4] Holbrook JA, Neu-Yilik G, Hentze MW, Kulozik AE. Nonsense-mediated decay approaches the clinic. Nature genetics. 2004;36(8):801–8. Epub 2004/07/31. 15284851 10.1038/ng1403

[5] Keeling KM, Wang D, Conard SE, Bedwell DM. Suppression of premature termination codons as a therapeutic approach. Critical reviews in biochemistry and molecular biology. 2012;47(5):444–63. Epub 2012/06/08. 10.3109/10409238.2012.694846 22672057 PMC3432268

[6] Bhuvanagiri M, Lewis J, Putzker K, Becker JP, Leicht S, Krijgsveld J, et al 5-azacytidine inhibits nonsense-mediated decay in a MYC-dependent fashion. EMBO molecular medicine. 2014;6(12):1593–609. Epub 2014/10/17. 10.15252/emmm.201404461 25319547 PMC4287977

[7] Keeling KM, Wang D, Dai Y, Murugesan S, Chenna B, Clark J, et al Attenuation of nonsense-mediated mRNA decay enhances in vivo nonsense suppression. PloS one. 2013;8(4):e60478 Epub 2013/04/18. 10.1371/journal.pone.0060478 23593225 PMC3622682

[8] Bidou L, Allamand V, Rousset JP, Namy O. Sense from nonsense: therapies for premature stop codon diseases. Trends in molecular medicine. 2012;18(11):679–88. Epub 2012/10/23. 10.1016/j.molmed.2012.09.008 23083810

[9] Salvatori F, Breveglieri G, Zuccato C, Finotti A, Bianchi N, Borgatti M, et al Production of beta-globin and adult hemoglobin following G418 treatment of erythroid precursor cells from homozygous beta(0)39 thalassemia patients. American journal of hematology. 2009;84(11):720–8. Epub 2009/10/08. 10.1002/ajh.21539 19810011 PMC3572903

[10] Forge A, Schacht J. Aminoglycoside antibiotics. Audiology & neuro-otology. 2000;5(1):3–22. Epub 2000/02/25. 10686428 10.1159/000013861

[11] Swan SK. Aminoglycoside nephrotoxicity. Seminars in nephrology. 1997;17(1):27–33. Epub 1997/01/01. 9000547

[12] Xue X, Mutyam V, Tang L, Biswas S, Du M, Jackson LA, et al Synthetic aminoglycosides efficiently suppress cystic fibrosis transmembrane conductance regulator nonsense mutations and are enhanced by ivacaftor. American journal of respiratory cell and molecular biology. 2014;50(4):805–16. Epub 2013/11/21. 10.1165/rcmb.2013-0282OC 24251786 PMC4068923

[13] Shulman E, Belakhov V, Wei G, Kendall A, Meyron-Holtz EG, Ben-Shachar D, et al Designer aminoglycosides that selectively inhibit cytoplasmic rather than mitochondrial ribosomes show decreased ototoxicity: a strategy for the treatment of genetic diseases. The Journal of biological chemistry. 2014;289(4):2318–30. Epub 2013/12/05. 10.1074/jbc.M113.533588 24302717 PMC3900975

[14] Nudelman I, Rebibo-Sabbah A, Cherniavsky M, Belakhov V, Hainrichson M, Chen F, et al Development of novel aminoglycoside (NB54) with reduced toxicity and enhanced suppression of disease-causing premature stop mutations. Journal of medicinal chemistry. 2009;52(9):2836–45. Epub 2009/03/25. 10.1021/jm801640k 19309154 PMC2832307

[15] Gunn G, Dai Y, Du M, Belakhov V, Kandasamy J, Schoeb TR, et al Long-term nonsense suppression therapy moderates MPS I-H disease progression. Molecular genetics and metabolism. 2014;111(3):374–81. Epub 2014/01/15. 10.1016/j.ymgme.2013.12.007 24411223 PMC3943726

[16] Auld DS, Lovell S, Thorne N, Lea WA, Maloney DJ, Shen M, et al Molecular basis for the high-affinity binding and stabilization of firefly luciferase by PTC124. Proceedings of the National Academy of Sciences of the United States of America. 2010;107(11):4878–83. Epub 2010/03/03. 10.1073/pnas.0909141107 20194791 PMC2841876

[17] Auld DS, Thorne N, Maguire WF, Inglese J. Mechanism of PTC124 activity in cell-based luciferase assays of nonsense codon suppression. Proceedings of the National Academy of Sciences of the United States of America. 2009;106(9):3585–90. Epub 2009/02/12. 10.1073/pnas.0813345106 19208811 PMC2638738

[18] McElroy SP, Nomura T, Torrie LS, Warbrick E, Gartner U, Wood G, et al A lack of premature termination codon read-through efficacy of PTC124 (Ataluren) in a diverse array of reporter assays. PLoS biology. 2013;11(6):e1001593 Epub 2013/07/05. 10.1371/journal.pbio.1001593 23824517 PMC3692445

[19] Welch EM, Barton ER, Zhuo J, Tomizawa Y, Friesen WJ, Trifillis P, et al PTC124 targets genetic disorders caused by nonsense mutations. Nature. 2007;447(7140):87–91. Epub 2007/04/24. 17450125 10.1038/nature05756

[20] Du L, Damoiseaux R, Nahas S, Gao K, Hu H, Pollard JM, et al Nonaminoglycoside compounds induce readthrough of nonsense mutations. The Journal of experimental medicine. 2009;206(10):2285–97. Epub 2009/09/23. 10.1084/jem.20081940 19770270 PMC2757881

[21] Du L, Jung ME, Damoiseaux R, Completo G, Fike F, Ku JM, et al A new series of small molecular weight compounds induce read through of all three types of nonsense mutations in the ATM gene. Molecular therapy: the journal of the American Society of Gene Therapy. 2013;21(9):1653–60. Epub 2013/06/19. 23774824 10.1038/mt.2013.150 PMC3776636

[22] Gonzalez-Hilarion S, Beghyn T, Jia J, Debreuck N, Berte G, Mamchaoui K, et al Rescue of nonsense mutations by amlexanox in human cells. Orphanet journal of rare diseases. 2012;7:58 Epub 2012/09/04. 10.1186/1750-1172-7-58 22938201 PMC3562214

[23] Taguchi A, Hamada K, Kotake M, Shiozuka M, Nakaminami H, Pillaiyar T, et al Discovery of natural products possessing selective eukaryotic readthrough activity: 3-epi-deoxynegamycin and its leucine adduct. ChemMedChem. 2014;9(10):2233–7. Epub 2014/07/22. 10.1002/cmdc.201402208 25044534

[24] Bidou L, Hatin I, Perez N, Allamand V, Panthier JJ, Rousset JP. Premature stop codons involved in muscular dystrophies show a broad spectrum of readthrough efficiencies in response to gentamicin treatment. Gene therapy. 2004;11(7):619–27. Epub 2004/02/20. 14973546 10.1038/sj.gt.3302211

[25] Grentzmann G, Ingram JA, Kelly PJ, Gesteland RF, Atkins JF. A dual-luciferase reporter system for studying recoding signals. RNA. 1998;4(4):479–86. Epub 1998/06/18. 9630253 PMC1369633

[26] Bidou L, Stahl G, Hatin I, Namy O, Rousset JP, Farabaugh PJ. Nonsense-mediated decay mutants do not affect programmed -1 frameshifting. RNA. 2000;6(7):952–61. Epub 2000/08/05. 10917592 10.1017/s1355838200000443 PMC1369972

[27] Keeling KM, Lanier J, Du M, Salas-Marco J, Gao L, Kaenjak-Angeletti A, et al Leaky termination at premature stop codons antagonizes nonsense-mediated mRNA decay in S. cerevisiae. RNA. 2004;10(4):691–703. Epub 2004/03/24. 15037778 10.1261/rna.5147804 PMC1262634

[28] Harger JW, Dinman JD. Evidence against a direct role for the Upf proteins in frameshifting or nonsense codon readthrough. RNA. 2004;10(11):1721–9. Epub 2004/09/25. 15388879 10.1261/rna.7120504 PMC1236997

[29] Keppler-Ross S, Noffz C, Dean N. A new purple fluorescent color marker for genetic studies in Saccharomyces cerevisiae and Candida albicans. Genetics. 2008;179(1):705–10. Epub 2008/05/22. 10.1534/genetics.108.087080 18493083 PMC2390648

[30] Bidou L, Rousset JP, Namy O. Translational errors: from yeast to new therapeutic targets. FEMS yeast research. 2010;10(8):1070–82. Epub 2010/10/20. 10.1111/j.1567-1364.2010.00684.x 20955199 PMC7110152

[31] Namy O, Hatin I, Rousset JP. Impact of the six nucleotides downstream of the stop codon on translation termination. EMBO reports. 2001;2(9):787–93. Epub 2001/08/25. 11520858 10.1093/embo-reports/kve176 PMC1084031

[32] Altamura N, Castaldo R, Finotti A, Breveglieri G, Salvatori F, Zuccato C, et al Tobramycin is a suppressor of premature termination codons. Journal of cystic fibrosis: official journal of the European Cystic Fibrosis Society. 2013;12(6):806–11. Epub 2013/04/02. 23540394 10.1016/j.jcf.2013.02.007

[33] Bedwell DM, Kaenjak A, Benos DJ, Bebok Z, Bubien JK, Hong J, et al Suppression of a CFTR premature stop mutation in a bronchial epithelial cell line. Nature medicine. 1997;3(11):1280–4. Epub 1997/11/14. 9359706 10.1038/nm1197-1280

[34] Floquet C, Deforges J, Rousset JP, Bidou L. Rescue of non-sense mutated p53 tumor suppressor gene by aminoglycosides. Nucleic acids research. 2011;39(8):3350–62. Epub 2010/12/15. 10.1093/nar/gkq1277 21149266 PMC3082906

[35] Floquet C, Rousset JP, Bidou L. Readthrough of premature termination codons in the adenomatous polyposis coli gene restores its biological activity in human cancer cells. PloS one. 2011;6(8):e24125 Epub 2011/09/13. 10.1371/journal.pone.0024125 21909382 PMC3166079

[36] He F, Jacobson A. Nonsense-Mediated mRNA Decay: Degradation of Defective Transcripts Is Only Part of the Story. Annual review of genetics. 2015;49:339–66. Epub 2015/10/06. 10.1146/annurev-genet-112414-054639 26436458 PMC4837945

[37] Wang W, Czaplinski K, Rao Y, Peltz SW. The role of Upf proteins in modulating the translation read-through of nonsense-containing transcripts. The EMBO journal. 2001;20(4):880–90. Epub 2001/02/17. 11179232 10.1093/emboj/20.4.880 PMC145432

[38] Maderazo AB, He F, Mangus DA, Jacobson A. Upf1p control of nonsense mRNA translation is regulated by Nmd2p and Upf3p. Molecular and cellular biology. 2000;20(13):4591–603. Epub 2000/06/10. 10848586 10.1128/mcb.20.13.4591-4603.2000 PMC85857

[39] Lentini L, Melfi R, Di Leonardo A, Spinello A, Barone G, Pace A, et al Toward a rationale for the PTC124 (Ataluren) promoted readthrough of premature stop codons: a computational approach and GFP-reporter cell-based assay. Molecular pharmaceutics. 2014;11(3):653–64. Epub 2014/02/04. 10.1021/mp400230s 24483936 PMC4167060

[40] Halvey PJ, Liebler DC, Slebos RJ. A Reporter System for Translational Readthrough of Stop Codons in Human Cells. FEBS open bio. 2012;2:56–9. Epub 2012/05/09. 22563532 10.1016/j.fob.2012.04.004 PMC3342693

[41] Rakauskaite R, Liao PY, Rhodin MH, Lee K, Dinman JD. A rapid, inexpensive yeast-based dual-fluorescence assay of programmed—1 ribosomal frameshifting for high-throughput screening. Nucleic acids research. 2011;39(14):e97 Epub 2011/05/24. 10.1093/nar/gkr382 21602263 PMC3152369

[42] Goldmann T, Overlack N, Moller F, Belakhov V, van Wyk M, Baasov T, et al A comparative evaluation of NB30, NB54 and PTC124 in translational read-through efficacy for treatment of an USH1C nonsense mutation. EMBO molecular medicine. 2012;4(11):1186–99. Epub 2012/10/03. 10.1002/emmm.201201438 23027640 PMC3494875

[43] Wang D, Belakhov V, Kandasamy J, Baasov T, Li SC, Li YT, et al The designer aminoglycoside NB84 significantly reduces glycosaminoglycan accumulation associated with MPS I-H in the Idua-W392X mouse. Molecular genetics and metabolism. 2012;105(1):116–25. Epub 2011/11/08. 10.1016/j.ymgme.2011.10.005 22056610 PMC3253910

[44] Langedijk J, Mantel-Teeuwisse AK, Slijkerman DS, Schutjens MH. Drug repositioning and repurposing: terminology and definitions in literature. Drug discovery today. 2015. Epub 2015/05/16. 10.1016/j.drudis.2015.05.001 25975957

[45] He F, Ganesan R, Jacobson A. Intra- and intermolecular regulatory interactions in Upf1, the RNA helicase central to nonsense-mediated mRNA decay in yeast. Molecular and cellular biology. 2013;33(23):4672–84. Epub 2013/10/09. 10.1128/MCB.01136-13 24100012 PMC3838015

[46] He F, Jacobson A. Upf1p, Nmd2p, and Upf3p regulate the decapping and exonucleolytic degradation of both nonsense-containing mRNAs and wild-type mRNAs. Molecular and cellular biology. 2001;21(5):1515–30. Epub 2001/03/10. 11238889 10.1128/MCB.21.5.1515-1530.2001 PMC86698

